# The Relations of Parental Autonomy Support, Parental Control, and Filial Piety to Chinese Adolescents’ Academic Autonomous Motivation: A Mediation Model

**DOI:** 10.3389/fpsyg.2021.724675

**Published:** 2021-08-27

**Authors:** Mingchun Guo, Long Wang, Jamin Day, Yanhan Chen

**Affiliations:** ^1^School of Psychology, Fujian Normal University, Fuzhou, China; ^2^School of Psychology, Faculty of Health and Behavioural Sciences, The University of Queensland, Brisbane, QLD, Australia; ^3^School of Creative Industries, College of Human and Social Futures, The University of Newcastle, Callaghan, NSW, Australia; ^4^School of Psychology, South China Normal University, Guangzhou, China

**Keywords:** autonomy support, parental control, filial piety, self-determination theory, academic autonomous motivation

## Abstract

This study attempted to examine the mediating role of filial piety in the relationships between parental autonomy support and control and Chinese adolescents’ academic autonomous motivation. A set of questionnaires were administered to 492 adolescent students at two senior high schools in Fuzhou, China. Confirmatory Factor Analysis and Structural Equation Modeling were employed to analyze the data. The results showed that reciprocal filial piety (RFP) fully mediated the relationships of parental autonomy support and behavioral control with adolescents’ academic autonomous motivation. RFP did not significantly mediate the relationship between psychological control and academic autonomous motivation. Comparatively, authoritarian filial piety (AFP) did not play a significant mediating role in the relationship between the three parenting dimensions and adolescents’ academic autonomous motivation. The findings provide a new perspective for understanding the relationship between parenting behaviors and Chinese adolescents’ academic autonomous motivation.

## Introduction

Autonomous motivation means that individuals engage in activities out of their own choice, volition, or values, which consists of two forms of motivation, including intrinsic motivation and identified motivation (Deci and Ryan, 1985; Ryan and Connell, 1989). Intrinsic motivation refers to individuals doing an activity for joy or pleasure, whereas identified motivation refers to individuals engaging in an activity because of their own values or goals (Deci and Ryan, 1985; Ryan and Connell, 1989). Individuals with autonomous motivation tend to enjoy what they are doing and persist in challenging situations (Ryan and Deci, 2000). Moreover, research has consistently shown that autonomous motivation for academic learning contributes significantly to students’ academic achievement and subjective well-being across different grades, subjects, and cultures (e.g., Ahmed and Bruinsma, 2006; Velki, 2011; Feri et al., 2016).

### Parental Autonomy Support and Control and Children’s Academic Motivation

In the past decades, researchers have paid much attention to the impact of parental autonomy support and parental control on children’s academic motivation. Reviews of previous research have shown that parental autonomy support characterized by respecting children’s viewpoints, allowing children to make their own choices and supporting children’s initiatives and problem-solving efforts is positively related to children’s academic intrinsic motivation, autonomous self-regulation and metacognitive skills, which in turn contribute to their academic performance (Grolnick, 2009; Pino-Pasternak and Whitebread, 2010). Conversely, parental control characterized by asserting their authority, directing children’s behavior and problem solving for children is linked to children’s academic extrinsic motivation, performance goal orientation and poor academic performance (Grolnick, 2009; Pino-Pasternak and Whitebread, 2010).

Researchers have distinguished two types of parental control: psychological control and behavioral control (Barber et al., 1994). Psychological control refers to parents’ intrusion into children’s psychological and emotional life by guilt induction, love withdrawal and authority assertion (Barber et al., 2005; Wang et al., 2007); Comparatively, behavioral control is conceptualized as parents controlling and managing children’s activities and behaviors in the physical world and providing children with needed guidance. Some research has found that psychological control was negatively related to children’s self-determined motivation and self-directed learning (Lee and Kwon, 2012), and contributed to children’s learned helplessness (Filippello et al., 2018). In contrast, Lee et al. (2012) found that parental behavioral control was positively associated with children’s self-regulation, which in turn contributed to their school adjustment and academic achievement.

Chinese parents have high expectations of children’s academic achievement (Leung and Shek, 2011), and have higher levels of home-based involvement in children’s education compared with European American parents (Huntsinger and Jose, 2009; Wu et al., 2013). It implies that Chinese parents play an important role in Chinese children’s academic lives. Although some research has examined the underlying mechanism in the relationship between parenting behaviors and Chinese children’s academic motivation using mediators such as personality (Guo and Wu, 2011) and self-efficacy (Sun et al., 2021), little research has investigated the mechanism from a Chinese cultural perspective. We argue that one important Chinese cultural factor, that is filial piety, can help to further understand the process by which parental autonomy support and parental control relate to adolescents’ academic autonomous motivation. Filial piety is a crucial value in Chinese culture and emphasized in Chinese family life (Yeh, 2003), which contributes to children’s academic motivation (Chow and Chu, 2007). However, more empirical research is needed to examine the role of filial piety in the relationship between parenting behaviors and Chinese adolescents’ academic autonomous motivation.

### Parenting and Children’s Filial Piety Toward Parents

Filial piety contains a set of rules and ideas around how children should treat their parents (Ho, 1996). The duties that filial piety requires children range from material to emotional support for parents, including taking care of elderly parents, showing respect, deference and compliance to parents as well as memorializing them after parents pass away (Yeh and Bedford, 2003).

Yeh (2003) and Yeh and Bedford (2003) have developed a dual model of filial piety, classifying it into two dimensions: reciprocal filial piety (RFP) and authoritarian filial piety (AFP). RFP refers to children showing respect and love, and supporting and caring for parents out of gratitude for their efforts in raising and taking care of them; AFP means that children suppress their own wishes and comply with parents’ wishes due to their seniority, and protect parents’ reputation and continue the family lineage to meet cultural expectations within Chinese society (Yeh, 2003; Yeh and Bedford, 2003). Filial piety is shaped and developed primarily via parenting, hence how parents interact with and care for their children influence children’s development of filial piety toward parents (Yeh and Bedford, 2004; Chen, 2014).

Indeed, some research has shown that authoritative parenting (Huang and Yeh, 2013; Chen, 2014) and supportive parenting (Chen et al., 2015) were associated with both RFP and AFP, whereas authoritarian parenting (Huang and Yeh, 2013; Chen, 2014) was only related to AFP. Huang and Yeh (2013) further found that adolescents experiencing authoritative parenting had gratitude for their parents, which in turn was related to their RFP toward parents. Moreover, adolescents experiencing authoritative parenting also felt committed to suppress their own wishes and comply with parents’ wishes (i.e., committed compliance), thus developing high levels of AFP as well. In contrast, under authoritarian parenting, children complied with parents only because of external forces such as parents’ authority or others’ judgment (i.e., situational compliance), and thus developed high levels of AFP. Therefore, the findings imply that there are two ways for children to develop high levels of AFP, one through positive parenting and the other through negative parenting.

### Filial Piety and Children’s Academic Motivation

A few studies have examined the relationship between filial piety and children’s academic motivation. Early research has demonstrated that adolescents’ filial piety could significantly predict their academic motivation (Chow and Chu, 2007; Hui et al., 2011), even after controlling for a series of parent and child variables. Chow and Chu (2007) pointed out that making efforts and achievement in learning is a common way for Chinese children to bring honor to their parents and to repay their parents’ efforts and sacrifice in raising them, which contributes to children’s high levels of academic motivation.

Further, Chen (2016) found that Hong Kong university students with high levels of RFP tended to be motivated to study based on their own interests and desire to learn knowledge and skills (i.e., mastery orientation), which in turn was associated with their better academic achievement. Comparatively, those with high levels of AFP were likely to adopt performance-approach and performance-avoidance goals, which subsequently contributed to better and worse academic achievement, respectively. Chen (2016) explained that students with AFP might tend to conduct filial behaviors to satisfy cultural role requirements for students, thus contributing to their development of performance-oriented goals.

### Theoretical Framework

According to self-determination theory (Ryan and Deci, 2000; Grolnick, 2009), parents can help to facilitate children’s autonomous motivation by meeting their basic psychologic needs, including needs for autonomy, competence, and relatedness. Further, Grolnick (2009) conceptualized three parenting dimensions, including parental autonomy support versus control, parental structure, and parental involvement, which can satisfy the three basic needs, respectively and thus promote children’s autonomous motivation, by facilitating children’s intrinsic motivation and increasing autonomy for extrinsic motivation. Although Grolnick (2009) conceptualized parental autonomy support and parental control as two opposite constructs of the same continuum, empirical research has demonstrated that autonomy support, behavioral control and psychological control are relatively independent (Wang et al., 2007). Therefore, this study treated them as three independent variables and examined their relationships with adolescents’ filial piety and academic autonomous motivation.

Integrating the existing findings (Huang and Yeh, 2013; Chen, 2016) into the framework of self-determination theory (Ryan and Deci, 2000; Grolnick, 2009), we speculate that parental autonomy support can satisfy Chinese adolescents’ autonomy need, so that they have genuine gratitude for parents and develop high levels RFP toward their parents; subsequently, these adolescents would experience themselves as active agents and tend to enjoy learning activities, thus enhancing their academic intrinsic motivation. Moreover, it can also facilitate adolescents’ autonomous integration of parents’ academic expectations into their own values, thus they are likely to develop academic identified motivation. Both academic intrinsic motivation and academic identified motivation are parts of academic autonomous motivation (Deci and Ryan, 1985; Ryan and Connell, 1989). Comparatively, parental psychological control cannot satisfy adolescents’ need for autonomy, hence they only develop high levels of AFP toward parents because of cultural requirements or fear of parental authority. Consequently, the adolescents would be less likely to experience as active agents in academic learning or integrate parents’ academic expectations into their own values, thus not developing academic autonomous motivation.

Regarding behavioral control, since parents control children without intrusion into their psychological world, but with respect and guidance, it can be seen as a kind of parental structure (Grolnick, 2009; Grolnick and Pomerantz, 2009). As such, parental behavioral control can help adolescents to understand “how to achieve success and avoid failure in school (i.e., have a sense of perceived control)” (Grolnick, 2009), thus meeting their need for competence. As a result, adolescents have gratitude for parents and develop high levels of RFP toward their parents. Adolescents with high levels of RFP also tend to feel competent in academic learning, which helps to develop their academic intrinsic motivation for learning. Moreover, they are also likely to internalize parents’ expectations into their own values, thus developing academic identified motivation. Therefore, RFP would also mediate the relationship between behavioral control and academic autonomous motivation.

Moreover, adolescents experiencing parental autonomy support and behavioral control might also feel committed to comply with parents’ wishes, thus developing AFP toward parents. Although adolescents with AFP might tend to learn due to their compliance with parents’ expectations rather than out of their own willingness, they are less likely to integrate parents’ expectations into their own beliefs. Therefore, we believe that AFP would not mediate the relationship between autonomy support/behavioral control and academic autonomous motivation.

### The Present Study

**FIGURE 1:**
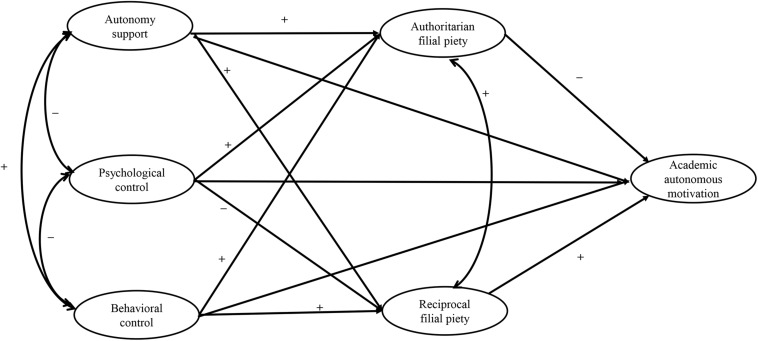
Conceptual model (+ refers to the positive relation and – refers to the negative relation).

This study aimed to examine the mediating role of filial piety in the relationships of parental autonomy support, psychological control, and behavioral control with Chinese adolescents’ academic autonomous motivation. Because senior high school students strive for autonomy and independence from parents at their age (Berk and Meyers, 2016), it is important to understand how parents can really facilitate their academic autonomous motivation. The hypothesized relationships among variables are presented in Figure 1.

## Methods

This study employed a cross-sectional research design and used convenience sampling for data collection. Because some researchers suggested at least five cases/observations per free parameters in a SEM (Structural Equation Modeling) model (Bentler and Chou, 1987; Bentler, 1995), with 63 free parameters in the SEM model, we tried to collect data from more than 315 adolescents for our study.

### Participants

Participants were 492 students in grades 10 and 11 from two senior high schools in Fuzhou, which is the capital city of Fujian Province in China. Forty-five students were removed from data analysis, because the participants: (1) selected the “disagree to participate” option on the informed consent form (*n* = 20); (2) completed questionnaires with no response variance (e.g., ticked one choice for the entire questionnaire; *n* = 17); or (3) did not fill out questionnaires (*n* = 8). Valid data were obtained from *N* = 447 students. Detailed demographic information is shown in Table 1.

**TABLE 1:** Demographic characteristics of the sample.

	N	%
**Child gender**		
Boys	205	45.86
Girls	241	53.91
**Single child or not**		
Yes	198	44.34
No	219	49.02
**Family status**		
Single-parent family	21	4.70
Intact family	420	93.96
**Multi-generation or nuclear family**		
Multi-generation family	186	41.61
Nuclear family	257	57.49
**Mother education**		
Elementary or lower	69	15.44
Junior middle school	129	28.86
Senior high school	92	20.58
Associate degree	71	15.88
Bachelor degree	67	14.99
Master or doctoral degree	10	2.24
**Father education**		
Elementary or lower	35	7.83
Junior middle school	122	27.29
Senior high school	113	25.28
Associate degree	57	12.75
Bachelor degree	97	21.70
Master or doctoral degree	16	3.58

*Note. Percentages may not add up to 100 due to missing data.*

### Procedure

All procedures were approved by the Academic Ethics Committee of Fujian Normal University. In this study, both adolescent participants and their parents read an information sheet and signed a consent form prior to participation. Nine different classes in the two senior high schools were randomly selected and 508 hardcopies of questionnaires were distributed to students in classrooms. Trained data collectors explained how to complete the questionnaires to students, and they received a notebook and a pen for completing the questionnaires. Finally, 492 students returned the questionnaires to data collectors and thus the response rate of the survey was 96.85%.

### Measures

#### Demographic Information

Demographic information was collected using a number of items asking participants about their gender, family status, number of siblings, father and mother education and etc.

#### The Parental Autonomy Support Scale

The Parental Autonomy Support Scale is a 12-item scale assessing perceived parental autonomy support (Wang et al., 2007). It has two dimensions including choice making, which has six items about parents allowing children to make their own choices/decisions (e.g., “My parents allow me to make choices whenever possible”), and opinion exchange, which consists of six items assessing the extent to which parents respect children’s opinions and exchange opinions with children (e.g., “My parents listen to my opinion or perspective when I’ve got a problem”). The items are rated on a 5-point scale. The measure has been used in a Chinese sample and obtained good concurrent validity and internal consistency (Chen et al., 2019).

#### The Psychological Control Scale

The Psychological Control Scale was used to measure perceived psychological control (Wang et al., 2007). It is an 18-item scale with three dimensions: guilt induction (10 items, e.g., “My parents tell me about all the things they have done for me”), love withdrawal (5 items, e.g., “My parents avoid looking at me when I have disappointed them”), and authority assertion (3 items, e.g., “My parents say, when I grow up, I will appreciate all the decisions they make for me”). Items are rated on a 5-point scale. The measure has shown adequate psychometric properties in a Chinese sample (Li et al., 2019).

#### The Behavioral Control Scale

The Behavioral Control Scale was used to assess perceived behavioral control (Wang et al., 2007). It is a 16-item scale including two factors, including solicitation (8 items, e.g., “My parents ask me to tell them what happens in school”) which assesses the extent to which parents ask or talk with children about their activities, friends and schoolwork and etc., and restriction (8 items, e.g., “My parents require me to speak with them before I decide on plans for weekends with my friends”) which measures parents’ restrictions on children’s activities and behaviors. The psychometric properties of this scale has been demonstrated among Chinese adolescents (Li et al., 2015). Participants are asked to rate how often their parents engage in a range of parenting practices on a 5-point scale.

#### The Dual Filial Piety Questionnaire

The Dual Filial Piety Questionnaire was used to assess children’s beliefs about RFP and AFP by asking participants to rate how important they think the ways children should treat their parents are for them (Yeh, 2003). It has two dimensions: AFP (8 items, e.g., “Live with parents even after marriage”) and RFP (8 items, e.g., “Talk with parents to know about their thoughts and feelings”). The psychometric properties of the measure has been demonstrated in Chinese samples (e.g., Chen and Ho, 2012). Participants are asked to respond to the items on a 6-point Likert scale.

#### The Chinese Motivated Strategies for Learning Questionnaire (MSLQ)

The Chinese Motivated Strategies for Learning Questionnaire (MSLQ) was used to assess students’academic motivation and self-regulated learning strategies (Rao and Sachs, 1999), which consists of two subscales: motivation subscale (22 items) and self-regulated learning subscale (22 items). The motivation subscale consists of three factors: intrinsic value, self-efficacy and test anxiety. Only the factor of intrinsic value was used in this study, because the items mainly assess students’ interest in learning (e.g., “I like what I am learning in school”) and their beliefs about the importance of learning (e.g., “It is important for me to learn what is being taught in this class”) (Pintrich and De Groot, 1990), which are actually student academic intrinsic moitvation and academic identified motivation. Moreover, intrinsic value has been found to be significantly related to students’ task-involved motivation, self-regulated learning strategies and academic achievement (e.g., Rao and Sachs, 1999; Rao et al., 2000). Therefore, we believe it can be used to measure academic autonomous motivation. The items are rated on a 7-point scale.

The CFA models of all the scales used in this study had good fit to the data, and Cronbach’s alpha coefficients ranged from 0.80 to 0.88. Detailed information about model fit indices and internal consistency can be obtained by contacting the corresponding author.

### Data Analysis

Using Harman’s single-factor test (Podsakoff et al., 2003), exploratory factor analysis was performed, and the results showed that the first factor explained 14.14% of total variance, indicating insignificant common method bias in this study. The proportion of missing data in the dataset was 0.43%. Full Information Maximum Likelihood (FIML) was used in Mplus 8.3 to handle missing data (Graham, 2009). The measurement model of the six latent constructs including the three parenting dimensions, RFP, AFP and academic autonomous motivation was examined by CFA and then the proposed mediation model was estimated using SEM in Mplus 8.3 (Muthén and Muthén, 1998-2017). The chi-square index (χ^2^), the root mean square error of approximation (RMSEA), the Tucker-Lewis index (TLI), the comparative fit index (CFI) and the standardized root mean square residual (SRMR) were used to assess the model fit. Model fit was deemed acceptable using the following cutoffs: TLI and CFI > 0.90 (Hu and Bentler, 1999; Marsh et al., 2004) and SRMR and RMSEA < 0.08 (Hu and Bentler, 1999; Browne and Draper, 2006).

Item parcels were created and used as indicators of latent variables to reduce model complexity and achieve good model fit (Little et al., 2002). For parental autonomy support, behaivoral control and psychological control, we used average scores of items for each dimension as indicators of latent variables according to the internal-consistency approach (Kishton and Widaman, 1994). Three parcels were created for dual filial piety and academic autonomous motivation based on the item-to-construct balance approach (Little et al., 2002). Because adolescent gender was significantly correlated with filial piety and academic autonomous motivation, it was included in the SEM model as a controlling variable. The mediation effects of filial piety was examined using the bias-corrected percentile bootstrap method with 1,000 resamples (Mackinnon, 2011). If the 95% CI (bias-corrected confidence intervals) of the indirect effects in the mediation model did not include zero, we considered the indirect effects to be statistically significant (Mackinnon, 2011).

## Results

### The Measurement Model

The results showed that the measurement model fit was satisfactory (χ^2^ = 307.71, *df* = 89, CFI = 0.94, TLI = 0.92, SRMR = 0.06, RMSEA = 0.07). The correlation matrix of latent constructs is shown in Table 2.

**TABLE 2:** Correlations, means and standard deviations of the study variables.

	***M***	***SD***	**1**	**2**	**3**	**4**	**5**	**6**	**7**	**8**	**9**	**10**	**11**	**12**	**13**	**14**
1. Child gender	—	0.50	—													
2. Child age	16.76	0.70	−0.12*	—												
3. Child grade	—	0.50	–0.07	0.73**	—											
4. Single child or not	—	0.50	0.16**	–0.06	–0.01	—										
5. Family status	—	0.21	0.04	0.02	0.02	–0.03	—									
6. Multi-generation or nuclear family	—	0.49	–0.05	–0.07	–0.05	–0.01	0.00	—								
7. Mother education	2.92	1.38	–0.02	–0.00	0.05	−0.33**	−0.10*	0.02	—							
8. Father education	3.24	1.36	–0.01	–0.06	–0.01	−0.35**	–0.02	–0.04	0.67**	—						
9. Autonomy support	3.43	0.75	0.06	0.09	0.13**	0.04	–0.03	0.05	0.08	0.04	—					
10. Psychological control	2.79	0.88	–0.09	–0.07	−0.13**	0.08	0.00	0.04	–0.09	–0.06	−0.47**	—				
11. Behavioral control	3.13	0.71	0.10*	−0.10*	–0.09	–0.01	–0.09	–0.02	0.07	–0.06	0.02	0.25**	—			
12. Authoritarian filial piety	2.82	0.89	−0.16**	0.08	0.06	0.02	–0.07	0.02	–0.07	–0.03	0.24**	0.10*	0.21**	—		
13. Reciprocal filial piety	5.01	0.71	0.15**	–0.03	–0.01	0.08	–0.01	0.01	–0.06	–0.06	0.41**	−0.13**	0.28**	0.29**	—	
14. Academic autonomous motivation	4.56	1.01	−0.21**	–0.07	–0.02	–0.06	–0.05	–0.01	0.06	0.08	0.20**	0.02	0.13**	0.16**	0.32**	—

Note. *p < 0.05. **p < 0.01; missing values were treated using the expectation maximization (EM) approach; the means of binary variables such as child gender are not presented.

### The Mediation Model

**FIGURE 2:**
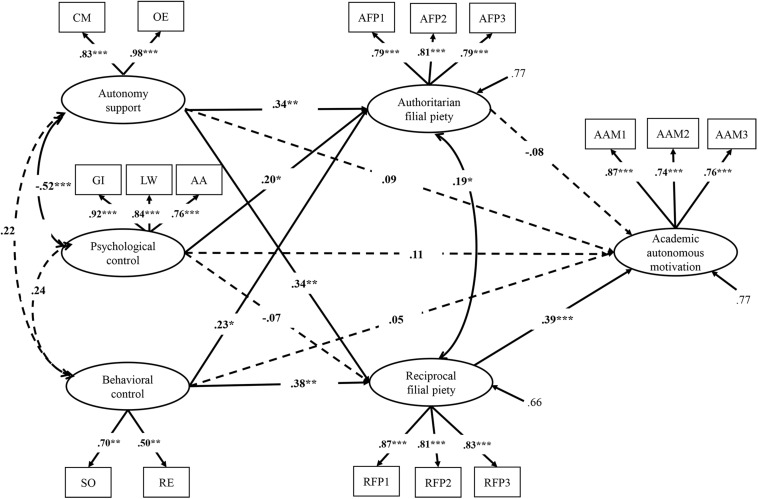
The mediation model of the study variables (Presented are standardized coefficients; CM, choice making; OE, opinion exchange; GI, guilt induction; LW, love withdrawal; AA, authority assertion; SO, solicitation; RE, restriction; AFP, Authoritarian filial piety; RFP, Reciprocal filial piety, and AAM, Academic autonomous motivation; *p < 0.05, **p < 0.01, ***p < 0.001).

The results showed that the mediation model obtained good fit indices, χ^2^ = 355.47, *df* = 102, CFI = 0.93, TLI = 0.91, RMSEA = 0.07, SRMR = 0.07. Standardized parameter estimates are presented in Figure 2. As the figure shows, both autonomy support and behavioral control were significantly and positively associated with RFP, which in turn was significantly related to academic autonomous motivation. The three parenting dimensions were positively associated with AFP, but AFP was not related to academic autonomous motivation. Finally, the direct paths from the three parenting constructs to academic autonomous motivation were all not significant.

In terms of the indirect effects, RFP had a full mediation effect in the relations of autonomy support (β = 0.13, *SE* = 0.05, 95% CI [0.01, 0.22]) and behavioral control (β = 0.15, *SE* = 0.06, 95% CI[0.04, 0.32]) to academic autonomous motivation. In contrast, AFP did not mediate the relations of the three parenting dimensions to autonomous motivation. Autonomy support (β = 0.19, *SE* = 0.10, 95% CI [0.05, 0.34]) and behavioral control (β = 0.18, *SE* = 0.10, 95% CI [0.03, 0.43]) had significant total effects on academic autonomous motivation, whereas psychological control did not have significant total effect on academic autonomous motivation (β = 0.07, *SE* = 0.09, 95% CI [−0.21, 0.21]). The mediation model explained 23.50% of the variance in academic autonomous motivation.

## Discussion

This study administered a set of questionnaires to Chinese adolescents to examine the mediating role of filial piety in the relationship between perceived parental autonomy support and control and adolescents’ academic autonomous motivation. The results showed that parental autonomy support and behavioral control were positively associated with adolescents’ RFP, which in turn contributed to their academic autonomous motivation. Moreover, RFP fully mediated the relationship between autonomy support and behavioral control and academic autonomous motivation. In addition, psychological control was significantly associated with AFP, which was not significantly related to adolescents’ academic autonomous motivation, and AFP did not significantly mediate the relationship between psychological control and academic autonomous motivation. It should also be noted that parental autonomy support and behavioral control were also positively related to adolescents’ AFP, but AFP did not significantly mediate the relationship between autonomy support and behavioral control and academic autonomous motivation. These results largely provide support for the hypothesized model.

With respect to the relationship between the three parenting constructs and adolescents’ filial piety, the results of the present study are consistent with the findings of the existing research, which showed significant relationships of children’s perceptions of authoritative parenting (Huang and Yeh, 2013; Chen, 2014) and supportive parenting (Chen et al., 2015) with both RFP and AFP, whereas perceived authoritarian parenting (Huang and Yeh, 2013; Chen, 2014) was only related to AFP. It is not surprising since parental autonomy support and behavioral control can be seen as part of authoritative parenting, which means that parents place appropriate demands on children while being responsive, warm, supportive and providing autonomy for children; and psychological control can be seen as part of authoritarian parenting because authoritarian parents have high demands on children but are parent-centered and do not respond to children’s psychological needs (Baumrind, 1967; Darling and Steinberg, 1993). These findings indicate that RFP have distinctive relationships with positive and negative parenting, but AFP relates to both positive and negative parenting statistically in a similar pattern.

Regarding the association of filial piety with academic autonomous motivation, the results showed that RFP but not AFP was related to adolescents’ academic autonomous motivation. Considering the similarity between mastery goal orientation and academic autonomous motivation, the results can be seen as consistent with Chen (2016)’s findings. RFP is based on warm, close parent-child relationships (Yeh and Bedford, 2004) and have their autonomy need satisfied (e.g., Zhou et al., 2020), thus adolescents with high levels of RFP tend to be interested in learning. In the meantime, they are also likely to integrate parents’ academic expectations into their own values. Hence these adolescents tend to develop academic autonomous motivation. In contrast, AFP is based on parent-child hierarchy (Yeh, 2006), Adolescents with high levels of AFP might be likely to see learning as fulfilling an obligation for their parents, so that they only learn to meet child and student role requirements rather than out of their own interest or values. Therefore, adolescents are unlikely to develop academic autonomous motivation.

In addition to the similar pattern of relationship between AFP and positive and negative parenting, previous research also found that AFP was related to both individuals’ positive and negative psycho-social functioning. For example, some research found that AFP was significantly associated with individuals’ social competence, life satisfaction (Yao and Wei, 2016) and performance-approach goal orientation (Chen, 2016), whereas other research revealed significant relationship between AFP and individuals’ maladaptive cognitions, internet addiction (Wei et al., 2019) and performance-avoidance goal orientation (Chen, 2016). Therefore, we speculate that there might be different types of AFP which have different relations to different parenting behaviors and children’s developmental outcomes.

In summary, this study attempted to integrate filial piety into the framework of self-determination theory and the findings can provide a new perspective for understanding the mechanism in the relationship between parenting and children’s academic motivation. Moreover, the results also indicate that psychologists and educators can probably enhance adolescents’ RFP and academic autonomous motivation by encouraging and guiding their parents to provide autonomy support and use behavioral control for their adolescent children. However, there are three limitations in this study, including the limited area for recruiting participants, using cross-sectional research design, and employing self-report measures. Future studies can test the model in other areas of China and other cultural contexts, use longitudinal research design and multiple-informant approach to remedy the limitations.

## Data Availability Statement

The original contributions presented in the study are included in the article/supplementary materials, further inquiries can be directed to the corresponding author/s.

## Ethics Statement

The studies involving human participants were reviewed and approved by the Academic Ethics Committee of Fujian Normal University. Written informed consent to participate in this study was provided by the participants’ legal guardian/next of kin.

## Author Contributions

MG designed the research, wrote, and revised the manuscript. LW analyzed the data and wrote the manuscript with MG. JD proofread and revised the manuscript. YC involved in research design and collected the data. All authors listed have made a substantial, direct and intellectual contribution to the work, and approved it for publication.

## Conflict of Interest

The authors declare that the research was conducted in the absence of any commercial or financial relationships that could be construed as a potential conflict of interest.

## Publisher’s Note

All claims expressed in this article are solely those of the authors and do not necessarily represent those of their affiliated organizations, or those of the publisher, the editors and the reviewers. Any product that may be evaluated in this article, or claim that may be made by its manufacturer, is not guaranteed or endorsed by the publisher.
